# Limited Biomarker Potential for IgG Autoantibodies Reactive to Linear Epitopes in Systemic Lupus Erythematosus or Spondyloarthropathy

**DOI:** 10.3390/antib13040087

**Published:** 2024-10-12

**Authors:** S. Janna Bashar, Zihao Zheng, Aisha M. Mergaert, Ryan R. Adyniec, Srishti Gupta, Maya F. Amjadi, Sara S. McCoy, Michael A. Newton, Miriam A. Shelef

**Affiliations:** 1 Department of Medicine, University of Wisconsin-Madison, Madison, WI 53705, USA; janna.bashar@gmail.com (S.J.B.); zihao.zheng@wisc.edu (Z.Z.);; 2 Department of Statistics, University of Wisconsin-Madison, Madison, WI 53706, USA; manewton@wisc.edu; 3 Department of Pathology and Laboratory Medicine, University of Wisconsin-Madison, Madison, WI 53705, USA; 4 Department of Biostatistics and Medical Informatics, University of Wisconsin-Madison, Madison, WI 53726, USA; 5 William S. Middleton Memorial Veterans Hospital, Madison, WI 53705, USA

**Keywords:** antibody, autoantibody, biomarker, systemic lupus erythematosus, spondyloarthropathy, peptide array

## Abstract

Background: Autoantibodies are commonly used as biomarkers in autoimmune diseases, but there are limitations. For example, autoantibody biomarkers have poor sensitivity or specificity in systemic lupus erythematosus and do not exist in the spondyloarthropathies, impairing diagnosis and treatment. While autoantibodies suitable for strong biomarkers may not exist in these conditions, another possibility is that technology has limited their discovery. The purpose of this study was to use a novel high-density peptide array that enables the evaluation of IgG binding to every possible linear antigen in the entire human peptidome, as well as a novel machine learning approach that incorporates ELISA validation predictability in order to discover autoantibodies that could be developed into sensitive and specific markers of lupus or spondyloarthropathy. Methods: We used a peptide array containing the human peptidome, several viral peptidomes, and key post-translational modifications (6 million peptides) to quantify IgG binding in lupus, spondyloarthropathy, rheumatoid arthritis, Sjögren’s disease, and control sera. Using ELISA data for 70 peptides, we performed a random forest analysis that evaluated multiple array features to predict which peptides might be good biomarkers, as confirmed by ELISA. We validated the peptide prediction methodology in rheumatoid arthritis and COVID-19, conditions for which the antibody repertoire is well-understood, and then evaluated IgG binding by ELISA to peptides that we predicted would be highly bound specifically in lupus or spondyloarthropathy. Results: Our methodology performed well in validation studies, but peptides predicted to be highly and specifically bound in lupus or spondyloarthropathy could not be confirmed by ELISA. Conclusions: In a comprehensive evaluation of the entire human peptidome, highly sensitive and specific IgG autoantibodies were not identified in lupus or spondyloarthropathy. Thus, the pathogenesis of lupus and spondyloarthropathy may not depend upon unique autoantigens, and other types of molecules should be sought as optimal biomarkers in these conditions.

## 1. Introduction

Autoantibodies, which can be pathogenic and diagnostic, are a hallmark of autoimmune diseases. For example, most people with rheumatoid arthritis have anti-citrullinated protein antibodies [1], which are the basis for the diagnostic anti-cyclic citrullinated peptide (CCP) test. However, the use of autoantibodies as biomarkers in autoimmune and inflammatory disease is imperfect. About 25% of people with rheumatoid arthritis are seronegative [2] for anti-CCP, as well as for rheumatoid factor, a less specific test that detects antibodies against the Fc region of IgG. In spondyloarthropathies (which include ankylosing spondylitis, psoriatic arthritis, and inflammatory bowel disease-associated arthritis), there are no autoantibodies used as diagnostic tests. A lack of autoantibodies in both conditions contributes to delays in diagnosis and treatment [3,4,5]. In systemic lupus erythematosus, a positive anti-nuclear antibody (ANA) test, which is essentially universal in lupus, is also common in other systemic autoimmune diseases, like rheumatoid arthritis [6] and Sjögren’s disease [7], as well as in many healthy individuals [8]. Tests that are more specific for lupus, like anti-Smith and anti-double-stranded DNA antibodies, have limited sensitivity [9,10]. Further, these tests cannot differentiate between patients who will have life-threatening lupus with nephritis versus mild disease. In addition to these diagnostic and prognostic dilemmas, gaps exist in our understanding of the pathophysiology of these diseases, which impairs the development of optimal therapeutics. Knowing the targets of autoantibodies could provide clues to pathogenesis. Thus, a full characterization of the repertoire of autoantigens might allow for the development of improved clinical tests as well as improved therapeutics based on a better understanding of pathophysiology.

In recent years, high-density arrays containing thousands to millions of antigens have emerged as a potential approach to autoantibody discovery [11,12,13]. However, array cost can limit sample size such that the high dimensionality and small sample size challenge the statistical methodology for selecting peptides that have different binding in different groups. Several statistical approaches in the area of large-scale testing have been developed to increase power while avoiding inflation of the false discovery rate (e.g., Benjamani–Hochberg [14], q-value [15], ash [16], and MixTwice [17]), but they are imperfect. To overcome statistical limitations as well as to enhance robustness, results from peptide arrays are often confirmed by enzyme-linked immunosorbent assay (ELISA). Moreover, ELISA is a commonly used method for clinical labs to detect antibody biomarkers. However, ELISA can be time consuming and labor intensive and thus not feasible for very large numbers of peptides. Therefore, it would be extremely valuable to have an analytical method that could accurately identify peptides that are more highly bound in one group than another with a very high rate of concordance with ELISA results.

In this manuscript, we use a high-density peptide array that contains the entire human peptidome as well as several viral peptidomes and key post-translational modifications, sera from subjects with lupus, spondyloarthropathy, rheumatoid arthritis, Sjögren’s disease, and controls, as well as a machine learning approach in search of previously undiscovered autoantibodies in lupus and spondyloarthropathy. The novelty of this approach is two-fold: (1) the inclusion of the entire human peptidome so that every possible linear human autoantigen can be assessed, and (2) a random forest methodology that considers not only array outcomes, but also how array outcomes can be translated into ELISA outcomes. The scientific importance of this study is that a better understanding of autoantibodies in lupus and spondyloarthropathy will provide key insights into disease pathogenesis and guide the development of improved biomarkers.

## 2. Materials and Methods

### 2.1. Human Subjects

Clinical data and sera from the following subjects were obtained from the University of Wisconsin Rheumatology Biorepository [18]: systemic lupus erythematosus meeting the 2012 Systemic Lupus International Collaborating Clinics classification (SLICC) criteria with a history of biopsy-proven lupus nephritis (n = 8 severe lupus) or no history of lupus nephritis (n = 8 mild lupus) [19]; spondyloarthropathy (n = 8 rheumatologist-diagnosed ankylosing spondylitis, psoriatic arthritis, or inflammatory bowel disease-associated arthritis with radiographic axial disease); rheumatoid arthritis meeting the American College of Rheumatology (ACR)/European League Against Rheumatism (EULAR) 2010 criteria [20] with either negative clinical testing for anti-CCP and rheumatoid factor (n = 8) or test results twice the upper limit of normal for both tests (n = 8); Sjögren’s disease meeting ACR/EULAR 2016 criteria [21] with positive (n = 8) or negative (n = 8) clinical testing for anti-SSA; and age- and sex-matched controls without autoimmune, inflammatory, or hematologic disease (n = 16).

### 2.2. Peptide Array

A high-density peptide array (6 million peptides) was designed that contained the entire human peptidome (16 amino acid peptides tiled at 2 amino acid intervals) [22] as well as 12–16 amino acid peptides derived from the following proteins in their native form, a form in which all arginines were replaced by citrullines, and a form in which all lysines were replaced by homocitrullines: 122 human proteins primarily known to be citrullinated in the rheumatoid joint [23], the proteomes of several viruses (human cytomegalovirus [UP000000938], Zaire ebolavirus [UP000007209], Reston ebolavirus [UP000007207], human parvovirus B19 [UP000006624], Epstein–Barr virus [UP000153037], Herpes simplex virus type 1 [UP000106517], Chikungunya virus [UP000000569], Zika virus [UP000054557], O’nyong’nyong virus [UP000007787, UP000008868, UP000008382], Sindbis virus [UP000006710], Ross River virus [UP000006579], and Mayaro virus [UP000007774]) as well as wheat, peanut, and bovine milk proteins and selected proteins from additional species (primarily Bordatella pertussis, Prevotella intermedia, and Prevotella copri). The array was constructed and IgG binding to the array peptides was quantified for the sera noted above by Roche Nimblegen (Madison, USA) as previously noted [11,12,22,23,24,25,26]. We have previously confirmed the high reproducibility of this array technology [23].

### 2.3. Enzyme-Linked Immunosorbent Assay

ELISA was performed as previously described [18] with minor modifications. ELISA plates were incubated with 0.1 μM of C-terminal biotinylated peptides (Peptide 2.0 Inc., Chantilly, VA, USA), serum diluted to 1:100, and bound IgG detected with mouse anti-human IgG (clone JDC-10, Southern Biotech, Birmingham, AL, USA). High reproducibility was obtained by ensuring that absorbance values matched for two separate ELISAs for each subject/peptide pair.

### 2.4. Random Forest and Statistical Analyses

Random forest [27], and its R implementation random Forest [28], was used to determine which array features might best predict peptides highly bound by IgG in specific diseases that could be confirmed by ELISA. The following array features were evaluated when considering the disease group versus control groups: signal fold difference, local false discovery rate (locFDR) by MixTwice [17], r-value (an empirical Bayes tool to rank effect size [29]), the proportion of subjects in the experimental (disease) group having signal intensity ≥3 standard deviations over the mean of the whole array (prop_exp_), the proportion of subjects in the control group having a signal intensity <3 standard deviations below the mean (prop_con_), as well as fold difference, locFDR, prop_exp_, and prop_con_ for the nearest neighbor (NN) peptides (averaged value for one peptide on either side of the peptide of interest). For the array evaluated in this study, μ + 3σ is 1047.4. The three variables for ELISA experimentation responses were array–ELISA correlation >0.6 (Spearman’s), ELISA fold change ≥5, and ELISA *p*-value ≤ 0.1 by Wilcoxon test. A consensus sequence was generated using MEME Suite [30].

ELISA data were compared using a Mann–Whitney test, and array–ELISA correlation using a Spearman correlation (Prism, GraphPad, Boston, MA, USA).

## 3. Results

To identify peptides that are highly and specifically bound in lupus and spondyloarthropathy as well as in other conditions, we evaluated serum IgG binding for subjects with lupus (with and without nephritis), spondyloarthropathy, rheumatoid arthritis (seronegative and seropositive), Sjögren’s disease (anti-SSA negative and positive), and matched controls using a high-density peptide array that contains the entire human peptidome, several viral peptidomes, and two key post-translational modifications (citrullination and homocitrullination). From this array experiment and previous array experiments [18,23], we selected 70 peptides based on a variety of criteria, performed ELISAs (some previously published [18,23]), and compared array and ELISA results. ELISA results sometimes agreed and sometimes did not agree with array results, and disagreement led to peptides that could not discriminate between the selected disease group and controls [18,23].

We then performed a random forest analysis that evaluated multiple array features (accounting for direct quantity of group comparison, statistical significance for multiplicity, ranking of effect size, raw signal filter, and consideration of neighboring overlapping peptides) to predict the reproducibility from array to ELISA as well as the difference between groups from both a direct quantity and inferential statistics angle, as outlined in the Materials and Methods section. We found that the averaged area under the curve value, a combined measure of sensitivity and specificity, from 500 times 70–30% cross-validation in predicting ELISA–array correlation, ELISA fold value, and ELISA *p*-value was 0.82, 0.81, and 0.80, respectively, with lower values for individual features (Table 1).

**Figure 1:**
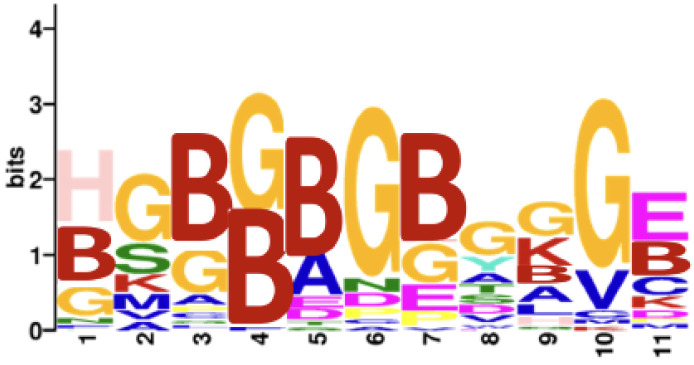
Motif for seropositive rheumatoid arthritis peptide antigens. A motif was obtained for the 1813 peptides predicted to be more highly bound by IgG in seropositive rheumatoid arthritis than controls; thresholded at 0.5 for all three predicted probabilities. B = citrulline.

As a validation step, we applied this methodology using the full array of 6 million peptides to compare seropositive rheumatoid arthritis (n = 8) versus age- and sex-matched controls (n = 8). After thresholding all three predicted probabilities by 0.5, 1813 peptides were identified. Consistent with the known citrulline dominance of the reactivity of rheumatoid arthritis autoantibodies [23], 99.1% of the selected peptides (1797 out of 1813) were citrulline-containing, while the proportion among all array peptides was only 3.1% citrulline-containing. Also, as shown in Figure 1, a motif of citrullines and glycines was found, consistent with previously described motifs [23,25,31]. These two observations support the validity of the methodology.

As a second and independent validation, we applied our methodology to a separate array study. To characterize antibody formation in response to SARS-CoV-2, a high-density peptide array was designed containing 389,128 overlapping peptides derived from SARS-CoV-2 and other viruses. Sixty subjects were evaluated (40 COVID-19-convalescent and 20 controls) with four array peptides validated by ELISA [32]. None of the peptides were included in the 70 peptides used to train the random forest learning model. As shown in Table 2, three of the four peptides had the desired ELISA results: correlation > 0.6, fold value ≥ 5, and *p*-value ≤ 0.1. If we had applied the same threshold rule as above, i.e., >0.5 in all predicted probabilities, we would have selected the three peptides that showed good empiric ELISA results and not the one peptide that did not have the desired fold value by ELISA or correlation between array and ELISA. Moreover, IgG binding to these three peptides, as measured by ELISA, allowed excellent discrimination between individuals who were COVID-19-convalescent and COVID-19-naïve [32], enabling their usage in future studies [33].

**Figure 2:**
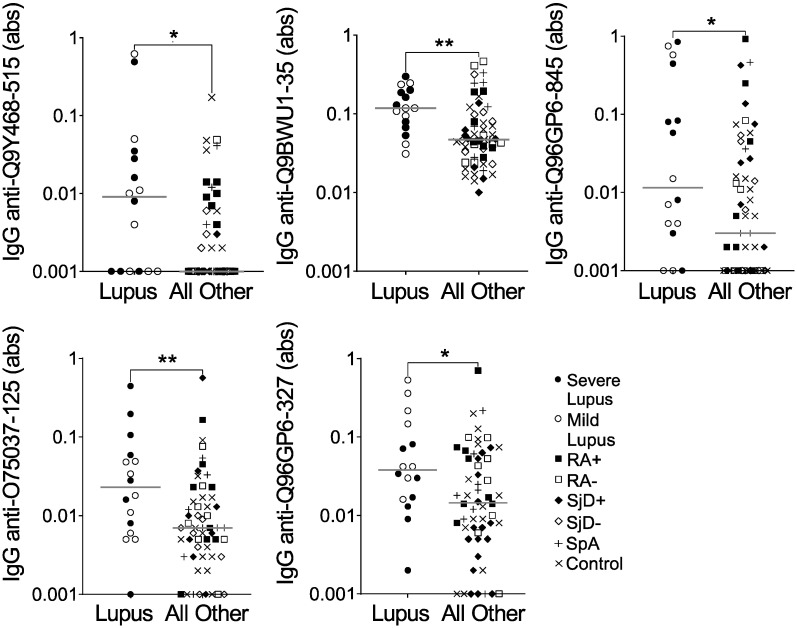
IgG binding to five peptides is modestly increased in lupus versus other diseases and controls. IgG binding to five peptides predicted to be highly bound in lupus was measured by ELISA for the same subjects used in the peptide array: lupus (SLE, n = 8 severe and n = 8 mild) and all other subjects (n = 56), including seropositive and seronegative rheumatoid arthritis (n = 8 RA+, n = 8 RA−), anti-SSA+ positive and negative Sjögren’s disease (n = 8 SjD+, n = 8 SjD−), spondyloarthropathy (SpA, n = 8), and non-autoimmune controls (n = 16). Absorbance (abs) values were compared for lupus versus all other subjects by Mann–Whitney test (* *p* < 0.05; ** *p* <0.01), and lines indicate medians.

Confident in the quality of our method of peptide selection, we moved forward using the same methodology to compare IgG binding to all 6 million array peptides for the 16 lupus subjects versus all other subjects on the array. We thresholded more conservatively so that the average of all three predicted probabilities was >0.75. We identified 289 peptides predicted to be highly bound, specifically in lupus, and then selected five peptides for validation by ELISA. As shown in Figure 2 and Table 3, all five peptides had a desired ELISA *p*-value of ≤0.1 (See Supplementary File S1). However, only one of the five peptides met the criteria of a fold value ≥5, and none of the five peptides met the correlation threshold of >0.6. Furthermore, IgG binding to all five peptides showed substantial overlap between lupus and all other subjects (Figure 2). Finally, although not part of the formal evaluation, we saw no obvious difference between severe and mild lupus for IgG binding to any peptide. Thus, although all five peptides were more highly bound by IgG in lupus than the other groups combined, they would not be good candidates for further use as biomarkers.

**Figure 3:**
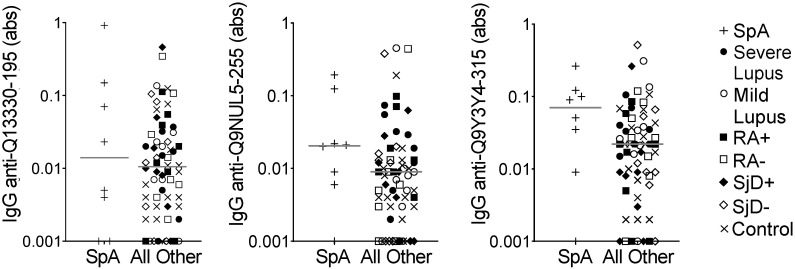
IgG binding to three peptides showed no difference in spondyloarthropathy versus other diseases and controls. IgG binding to three peptides predicted to be highly bound in spondyloarthropathy was measured by ELISA for the same subjects used in the peptide array: spondyloarthropathy (SpA, n = 8) and all other subjects (n = 64), including lupus (SLE, n = 8 severe and n = 8 mild), seropositive and seronegative rheumatoid arthritis (n = 8 RA+, n = 8 RA−), anti-SSA+ positive and negative Sjögren’s disease (n = 8 SjD+, n = 8 SjD−), and non-autoimmune controls (n = 16). Absorbance (abs) values were compared for SpA versus all other subjects by Mann–Whitney test (no significantly different comparisons), and lines indicate medians.

We then used the same model to evaluate the eight spondyloarthropathy subjects compared to all other subjects on the array. We identified only 13 peptides for which the average of all three predicted probabilities was >0.75. We selected three peptides for ELISA validation. Only one peptide met the *p*-value criteria of ≤0.1 and none of the peptides demonstrated the goal correlation or fold value parameters (Table 4). Also, as shown in Figure 3, there was substantial overlap in IgG binding between the spondyloarthropathy subjects and all other subjects. Thus, no peptides were identified as potential biomarkers for spondyloarthropathy.

## 4. Discussion

In this manuscript, we describe a novel method for identifying peptides highly bound by IgG using a relatively small number of samples and a high-density peptide array containing millions of peptides. Our analysis comprehensively accounted for direct quantity of group comparison, statistical significance for multiplicity, ranking of effect size, raw signal filter, and neighboring overlapping peptides. However, we did not identify novel highly bound peptides specific to either lupus or spondyloarthropathy.

One possible explanation for our inability to identify novel sensitive and specific autoantibodies in lupus or spondyloarthropathy with biomarker potential is methodological. Since our validation studies in COVID-19 and rheumatoid arthritis produced the expected results, the answer could lie in thresholding. Every selected peptide in the COVID-19 experiment had a probability of a *p*-value ≤ 0.1 of at least 0.97. Similarly, for rheumatoid arthritis peptides, 94% of the highly bound citrulline-containing peptides that met the 0.75 average probability cut-off (when comparing seropositive rheumatoid arthritis to all other groups apart from seronegative rheumatoid arthritis) had a probability for a *p*-value of ≤0.1 of at least 0.97. For the lupus and spondyloarthropathy peptides meeting the average 0.75 probability cut-off, there were no peptides with a probability for a *p*-value of ≤0.1 that met or exceeded 0.97. Further, when we selected human peptidome peptides more highly bound by IgG in lupus or spondyloarthropathy versus all other subjects, as reported for Sjögren’s disease [22], our lowest *p*-values were ~10^−4^ to 10^−5^, whereas the *p*-values in the COVID study [32] were 10^−12^ to 10^−22^, many orders of magnitude lower. Thus, our thresholds provided high sensitivity for identifying potentially highly bound peptides in lupus and spondyloarthropathy. However, even the most likely of these peptides did not have the extremely high probabilities seen for validated peptides in COVID-19 and RA, and then were not validated by ELISA. If we used more stringent thresholds, we would not have identified any peptides, suggesting that commonly and specifically highly bound linear peptides simply do not exist in these conditions.

In support of a lack of highly and specifically bound linear peptides in spondyloarthropathy, the few autoantigens that have been reported in psoriatic disease typically have high levels of overlap with other autoimmune and inflammatory conditions [34]. In contrast, using an array of 8087 human proteins [35], an anti-PPM1A autoantibody was discovered in ankylosing spondylitis that was not present in rheumatoid arthritis. Similarly, in a study of high-density nucleic acid programmable protein arrays expressing 3498 proteins, autoantigens were identified in ankylosing spondylitis that were not identified in rheumatoid arthritis [36]. We did not identify PPM1A or other autoantigens in our study, potentially due to our mix of spondyloarthropathy subjects, our use of multiple disease controls, or our inclusion of only linear peptides, which do not include conformational epitopes. However, although the anti-PPM1A autoantibody was used in a subsequent study [37], anti-PPM1A and other autoantibodies have not been developed into biomarkers, raising questions about sensitivity and specificity in large subject groups. Indeed, there are no autoantibody biomarkers in clinical use for the spondyloarthropathies, and thus highly specific autoantibodies simply may not exist. Consistent with this idea, there appear to be fundamental biological differences between autoantibody positive and negative arthritis [38,39].

We also could not identify and validate specific linear autoantigens in lupus. This result is similar to a study in which a phage-displayed library of 10^9^ random peptides with deep sequencing appeared to identify novel autoantigens, but the four peptides tested in ELISA did not show higher binding in lupus than in other diseases [40]. However, the use of a random peptide library followed by a peptide microarray of 44 peptides ultimately identified a panel of five autoantigens that were highly specific and sensitive for lupus [41]. This latter study also had a much larger sample size than ours [41]. Thus, a two-step approach with a large and then a small array and/or a larger sample size might be needed to identify autoantibodies.

Despite the lack of sensitive and specific autoantibodies in lupus and spondyloarthropathy, the combination of a high-density peptide array and random forest evaluation is promising in improving the identification of antibody and autoantibody repertoires in other autoimmune and inflammatory diseases, in infections, and in response to vaccines. This approach may be especially useful as methods are being developed to study the fine specificity of antibody responses to viruses like SARS-CoV-2 that mutate extensively, as well as to COVID-19 vaccines [42,43,44], particularly given the strong performance of our methodology with the COVID-19 array.

## 5. Conclusions

A combination of high-density peptide array and machine learning methodology is able to accurately identify peptides that are highly and specifically bound by IgG from an enormous pool of potential antigens. However, unique and highly bound autoantigens in lupus and spondyloarthropathy could not be found within the entire human peptidome, suggesting that these autoantibodies do not commonly exist. Thus, lupus and spondyloarthropathy pathogenesis is unlikely to depend upon unique autoantigens, and future development of biomarkers should consider markers that are not antibody-based.

## Figures and Tables

**Table 1:** Averaged area under the curve values for predicting ELISA outcomes for array features according to random forest analysis.

Array Features	ELISA-Array Coefficient	ELISA Fold Value	ELISA *p*-Value
Fold	0.72	0.74	0.72
Fold (NN) ^1^	0.68	0.75	0.67
r-value	0.64	0.57	0.70
locFDR ^2^	0.60	0.72	0.73
locFDR (NN)	0.63	0.65	0.65
prop_exp_ ^3^	0.64	0.72	0.71
prop_exp_ (NN)	0.67	0.73	0.71
prop_con_ ^4^	0.61	0.50	0.58
prop_con_ (NN)	0.64	0.50	0.59
All features	0.82	0.81	0.80

^1^ NN: nearest neighbor. ^2^ locFDR: local false discovery rate. ^3^ prop_exp_: proportion of subjects in the experimental group having signal intensity ≥3 standard deviations over the mean of the whole array. ^4^ prop_con_: proportion of subjects in the control group having a signal intensity <3 standard deviations below the mean of the whole array.

**Table 2:** ELISA results for SARS-CoV-2 peptides predicted or not predicted to be more highly bound by IgG in COVID-19-convalescent versus control sera using random forest methodology.

ID	Sequence	p^cor 1^	p^fold 2^	p*^p^*^-value 3^	E^cor 4^	E^fold 5^	E*^p^*^-value 6^
surf-1253	CCKFDEDDSEPVLKGV	0.63	0.60	0.98	0.83	8.75	2.3 × 10^−9^
surf-814	KRSFIEDLLFNKVTLA	0.55	0.44	0.98	0.59	1.98	6.0 × 10^−5^
mem-8	ITVEELKKLLEQWNLV	0.53	0.60	0.97	0.83	51.52	1.1 × 10^−8^
nucl-390	QTVTLLPAADLDDFSK	0.64	0.51	0.98	0.82	92.32	1.7 × 10^−9^

Values that do not meet criteria are red. ^1^ p^cor^: Predicted probability for obtaining an array-ELISA correlation coefficient >0.6. ^2^ p^fold^: Predicted probability for obtaining a fold value ≥5 by ELISA. ^3^ p*^p^*^-value^: Predicted probability for obtaining a *p*-value ≤ 0.1 by ELISA. ^4^ E^cor^: Empiric array-ELISA correlation coefficient. ^5^ E^fold^: Empiric fold value by ELISA. ^6^ E*^p^*^-value^: Empiric *p*-value by ELISA.

**Table 3:** ELISA results for peptides predicted to be more highly bound by IgG in lupus versus disease and non-disease control sera using random forest methodology.

ID	Sequence	p^cor 1^	p^fold 2^	p*^p^*^-value 3^	E^cor 4^	E^fold 5^	E*^p^*^-value 6^
Q9Y468-515	QPPLGPREPSSASPGG	0.89	0.89	0.84	0.44	9.3	0.013
Q9BWU1-35	VYKARRKDGKDEKEYA	0.80	0.88	0.83	0.52	1.6	0.003
Q96GP6-845	TPIQKPPRKKSREAAG	0.77	0.93	0.84	0.49	3.5	0.044
O75037-125	AERKRRAQEQGVAGPE	0.81	0.89	0.86	0.46	2.5	0.009
Q8TE54-327	AQGSAKKFKYSIDDNQ	0.95	0.88	0.80	0.36	2.2	0.025

Values that do not meet criteria are red. ^1^ p^cor^: Predicted probability for obtaining an array-ELISA correlation coefficient >0.6. ^2^ p^fold^: Predicted probability for obtaining a fold value ≥5 by ELISA. ^3^ p*^p^*^-value^: Predicted probability for obtaining a *p*-value ≤0.1 by ELISA. ^4^ E^cor^: Empiric array-ELISA correlation coefficient. ^5^ E^fold^: Empiric fold value by ELISA. ^6^ E*^p^*^-value^: Empiric *p*-value by ELISA.

**Table 4:** ELISA results for peptides predicted to be more highly bound by IgG in spondyloarthropathy versus disease and non-disease control sera using random forest methodology.

ID	Sequence	p^cor 1^	p^fold 2^	p*^p^*^-value 3^	E^cor 4^	E^fold 5^	E*^p^*^-value 6^
Q13330-195	ETQVWEAHNPLTDKQI	0.756	0.828	0.844	0.17	4.1	0.721
Q9NUL5-255	LSQGGLLEDLDNLILE	0.618	0.928	0.876	0.40	1.4	0.123
Q9Y3Y4-315	DACTTEKSNKSSLHPN	0.898	0.786	0.674	0.26	1.8	0.089

Values that do not meet criteria are red. ^1^ p^cor^: Predicted probability for obtaining an array-ELISA correlation coefficient >0.6. ^2^ p^fold^: Predicted probability for obtaining a fold value ≥5 by ELISA. ^3^ p*^p^*^-value^: Predicted probability for obtaining a *p*-value ≤0.1 by ELISA. ^4^ E^cor^: Empiric array–ELISA correlation coefficient. ^5^ E^fold^: Empiric fold value by ELISA. ^6^ E*^p^*^-value^: Empiric *p*-value by ELISA.

## Data Availability

The original contributions presented in the study are included in the supplementary material, further inquiries can be directed to the corresponding author.

## Supplementary Materials

The following supporting information can be downloaded at: https://www.mdpi.com/article/10.3390/antib13040087/s1, File S1: Array-ELISA values.
